# Lizards ran bipedally 110 million years ago

**DOI:** 10.1038/s41598-018-20809-z

**Published:** 2018-02-15

**Authors:** Hang-Jae Lee, Yuong-Nam Lee, Anthony R. Fiorillo, Junchang Lü

**Affiliations:** 1 Geological Museum, Korea Institute of Geoscience and Mineral Resources, Daejeon, 34123 South Korea; 2 School of Earth and Environmental Sciences, Seoul National University, Seoul, 08826 South Korea; 3 Perot Museum of Nature and Science, Dallas, TX 75201 United States; 4 Institute of Geology, Chinese Academy of Geological Sciences, Beijing, 100037 China

## Abstract

Four heteropod lizard trackways discovered in the Hasandong Formation (Aptian-early Albian), South Korea assigned to *Sauripes hadongensis*, n. ichnogen., n. ichnosp., which represents the oldest lizard tracks in the world. Most tracks are pes tracks (N = 25) that are very small, average 22.29 mm long and 12.46 mm wide. The pes tracks show “typical” lizard morphology as having curved digit imprints that progressively increase in length from digits I to IV, a smaller digit V that is separated from the other digits by a large interdigital angle. The manus track is 19.18 mm long and 19.23 mm wide, and shows a different morphology from the pes. The predominant pes tracks, the long stride length of pes, narrow trackway width, digitigrade manus and pes prints, and anteriorly oriented long axis of the fourth pedal digit indicate that these trackways were made by lizards running bipedally, suggesting that bipedality was possible early in lizard evolution.

## Introduction

Although lizards are the most successful of modern reptiles in terms of the number of species (more than 5,800 extant species) and their wide geographical distribution^1^, their fossil record is relatively poor with respect to both skeletons and tracks. It is, in general, because their small bodies require a suitable depositional environment for preservation. Crown-group Squamata originated between the Late Triassic and the Early Jurassic (213~176 Ma) based on molecules and fossils^2^. Some skeletal materials of Iguania, Gekkota, Scincoidea, Lacertoidea, and Anguimorpha have been reported in Asia, Europe, and North America by the Early Cretaceous^3^. Unfortunately, fossil footprint records attributable to lizards are even rarer than those of body fossils because of the general light body weight of lizards and their preferred range of habitats^4^. There are thus far only three previous reports of fossil lizard tracks. Two were attributed to a lizard without a description from the Eocene Green River Formation, Utah: one is a lizard trackway^5,6^ and the other is one isolated track^7^. A new lizard trackway, *Neosauroides koreaensis*, was recently named from the Haman Formation (Late Albian~Early Cenomanian) of South Korea^8^.

Here we describe the oldest crown-group lizard trackways known anywhere in the world which show bipedal locomotion. Tracks come from the Hasandong Formation (Aptian~early Albian)^9^, South Korea which are extraordinarily well-preserved and allow identification of detailed foot anatomy. Associated with a complete manus imprint, most tracks are very small (less than 26 mm) and appear as ectaxonic (i.e., longer fourth digit) and asymmetrical imprints which are frequently recognized as a diagnostic feature of the “typical” lizard pes^10^. Many modern lizards can bipedally run on the land and even on the surface of water (e.g., the “Jesus lizard”, *Basiliscus basiliscus*)^11^. However, it has been unclear as to when lizards developed a capability for bipedal locomotion, though bipedal locomotion has been inferred in some fossil lizards based on the relationship between forelimb and hind limb lengths (e.g., *Tijubina pontei*)^12,13^. Therefore, the discovery described in this report is highly significant because it is the first direct evidence of bipedal locomotion in fossil lizards, suggesting that lizard bipedality is deeply rooted in the phylogeny of lizard evolution.

## Results

### Systematic ichnology

Order Squamata Oppel, 1811

*Sauripes hadongensis* ichnogen. et ichnosp. nov.

### Etymology

Ichnogenus named from ancient Greek “sauros” (lizard) and “pes” (foot). Ichnospecies named after Hadong County that yielded the holotype.

### Holotype

Manus and pes prints on a mudstone slab (70 × 30 cm) (KIGAM VP 201501: Korea Institute of Geoscience and Mineral Resources, Vertebrate Paleontology).

### Type locality and horizon

Hasandong Formation, Lower Cretaceous (Aptian-early Albian)^9^, an abandoned quarry next to Hadong power plant, Hadong County, South Gyeongsang Province, South Korea (Supplementary Information Fig. S1).

### Diagnosis

Quadrupedal tracks; manus prints are medial to the pes prints; the pes prints are larger than the manus prints; plantigrade and pentadactyl pes prints are longer than wide; the digit length progressively increasing from digits I to IV (ectaxonic); digit V is oriented more laterally and offset from other digits; digit imprint IV is more than twice the length of the metatarsal impression; plantigrade and pentadactyl manus print has similar length and width dimensions; digits II and IV are shorter than digit III (mesaxonic); the interdigital angle between digits I and V of the manus is larger than that of the pes.

### Description

**Figure 1:**
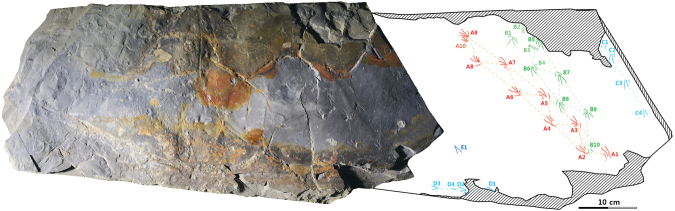
Photograph and drawing of lizard trackways on the block.

The slab contains 29 lizard tracks without any other vertebrate traces (Fig. 1). They are preserved as depressions and are not underprints. Although the tracks are shallowly depressed and very small in size, the quality of the impressions of the autopod anatomy in some tracks is good enough to provide detailed descriptions.

Based on track morphology, two different types of tracks are observed on this slab. One type (N = 25, pes tracks) has curved digit imprints that progressively increase in length from digits I to IV, a smaller digit V that is distinctly separated from the other digits by a large interdigital angle, and digit V is oriented more laterally. The other type (N = 4, manus tracks) is mesaxonic, having a longer digit III compared to the others (digits I, II, IV, V). The average manus and pes length is 19.18 mm and 22.29 mm, respectively (heteropody). Although manus-pes sets are not regularly imprinted, the tracks visible on the slab clearly show locomotion patterns without tail trails.

**Figure 2:**
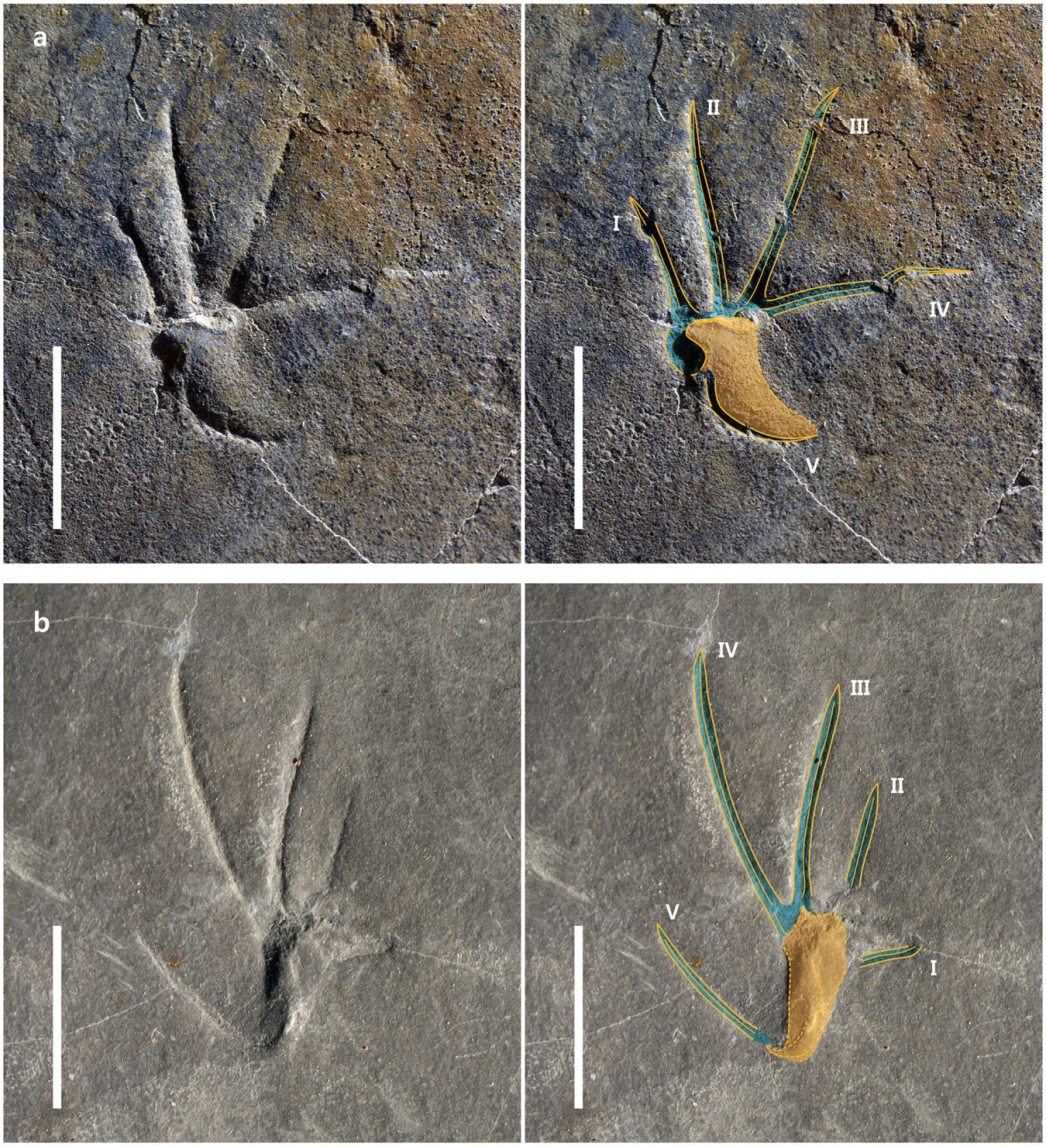
Manus and pes tracks of *Sauripes hadongensis*, n. ichnogen., n. ichnosp. (**a)** Enlarged photograph and drawing of a manus imprint (B1). (**b)** A pes imprint (A6). Scale bars equal 1 cm.

Of the four manus tracks, one in trackway B (B1, Fig. 2a) is better preserved than the other manus tracks in trackway A (A10) and trackway B (B3, B4). It has five digits which appear nearly straight except for digit V which is strongly curved medially. All digit impressions are very narrow (less than 1 mm). The digit I (7.58 mm long) and II (11.91 mm long) imprints are anteromedially oriented, whereas those of digit III (13.64 mm long) are oriented anterolaterally, digit IV (12.69 mm long) laterally, and digit V (6.84 mm long) posterolaterally. Therefore, the divarication of digit I and V impressions is very wide (134.42°). The distal ends of digit III and IV show slightly curved claw marks. The metacarpal depression is small and slightly raised compared to the digital impressions. There is no indication of webbing between the digits.

**Figure 3:**
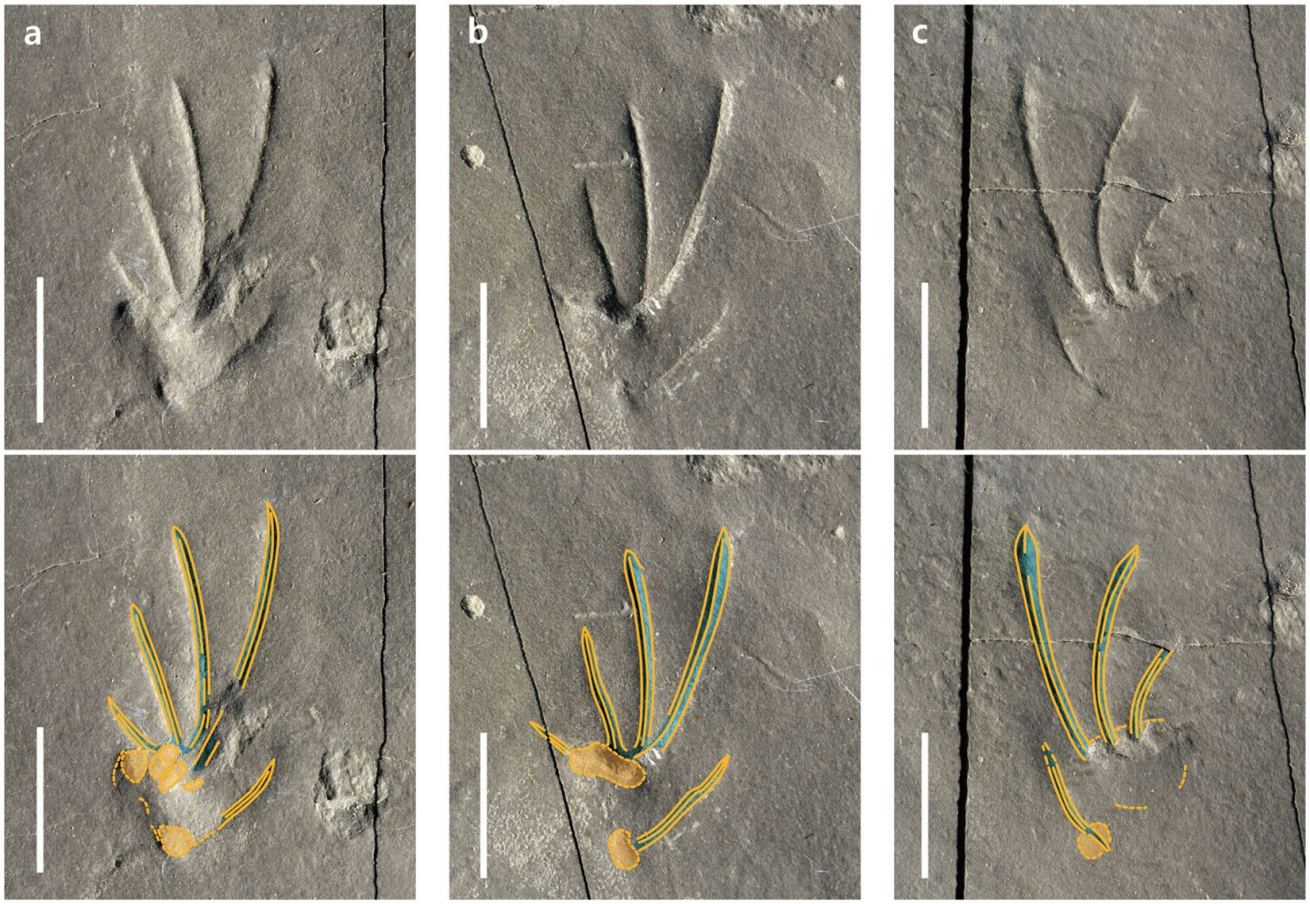
Pes tracks of *Sauripes hadongensis*, n. ichnogen., n. ichnosp. (**a)** Enlarged photograph and drawing of a pes imprint (A3). (**b)** A pes imprint (B8). (**c**) A pes imprint (B9). Scale bars equal 1 cm.

The pes prints are plantigrade, pentadactyl, and distinctly ectaxonic (Figs 2b, 3). Digit I imprint is the shortest (average 4.34 mm) and digit II, III, and IV imprints (7.07 mm, 12.84 mm, and 15.80 mm, respectively) increase progressively in length. Digit V (9.48 mm) is shorter than III and IV, but longer than I and II. Digit I imprint is oriented medially (A6, Fig. 2b) or anteromedially (A3, B8, Fig. 3a,b). Digit V imprint is distinctly separated from other imprints and is connected to the back of the heel trace. The divarication of digit I and V impressions is less than 90°. The metatarsal impression is elongated and located behind digits I to IV. It is slightly raised in relation to the digit imprints, with the metatarsophalangeal joint area the most deeply impressed, especially in digits II, III, and IV.

**Figure 4:**
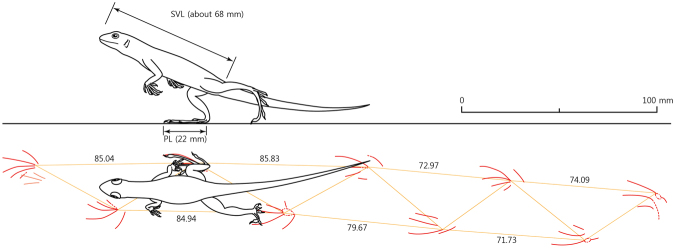
A reconstruction of a lizard bipedal running on the substrate, based on trackway A. Abbreviations: SVL, snout-vent length; PL, pes length.

Twenty-eight tracks comprise four trackways, and the trackways have roughly two directions (Fig. 1). Trackways A and B slightly overlap in the opposite direction, indicating a short time interval between two formations. They consist of ten left and right tracks, respectively, while trackways C and D preserve four right tracks, respectively, on the slab (Supplementary Information Table S1). Pes prints are predominant in all trackways, so it is not easy to recognize a normal quadrupedal gait pattern, comprising manus and pes prints. The manus gait-width is narrower than the pes gait-width as seen in the trackways of extant lacertids^14^ and varanids^15^. Trackway A is the longest and best preserved among the four trackways, comprising one incomplete right manus and nine pes prints (4 left and 5 right). The average pes stride length is 79.18 mm and the average pace length is 47.82 mm with 112.88° as pace angulation. The trackway width becomes narrower as stride length increases in trackways A and B (Fig. 4). The snout-vent length (SVL) of the trackmaker was approximately 68 mm, based on the allometric plot for snout-vent length in relation to foot length of an iguanian *Tropidurus torquatus*^16^.

## Discussion

### Bipedality of *Sauripeshadongensis*

Many extant lizard species can run bipedally, but not as obligate bipedality. Lizards exhibit different gaits, from quadrupedal and bipedal species to terrestrial and arboreal specialists. Lizard locomotion is strongly influenced by body shape and length, as well as by differences in habitat^17^. Nevertheless, four forms of gait; a quadrupedal walk at low speeds, a quadrupedal fast gait, a diagonal run at high speeds, and the bipedal run (e.g., *Basiliscus basiliscus*) are recognized for locomotion in lizards^18^. Many lizards (more than 50 species) are known to have the capability for bipedal locomotion^19^. Although some lizards appear to run bipedally without acceleration^19^, bipedality usually occurs as a consequence of acceleration in a lizard with hind limbs that are significantly longer than the forelimbs, moving the center of mass, and the rotational force on the hip joints^20,21^. When lizards are moving at relatively slow speeds, they retain a sprawling limb posture with laterally oriented plantigrade feet^22^. With this locomotor pattern, the front feet are positioned under the body with the head up, increasing the chance of leaving manus imprints rather than pes ones as seen in *Neosauroides koreanesis*^8^. In contrast, *Sauripes hadongensis* shows pes-dominant trackways characterized by long strides, a large pace angulation, and digitigrade prints throughout the locomotion sequences. Snyder pointed out that long hind limbs, short forelimbs, a narrow pelvis and a long tail could aid bipedality in lizards, mostly through increased stride length^23^. Running lizards at high speeds frequently also leave digitigrade footprints rather than plantigrade ones^24^.

*S*. *hadongensis* has better defined impressions of the digits than of the sole pads, with distinctly deep metatarsophalangeal joint in digits II, III, and IV, behind which the sediment is slightly pushed up, indicating that they ran mainly on the digits, instead of touching the whole soles plantar surface on the substrate (Fig. 3). At fast speeds, the long axis of the fourth toe is nearly parallel to the direction of movement, generating a great proportion of the forces^25^, as is clearly shown in trackway A and B (Fig. 1). However, the extant and fossil lizard pes prints in walking trackways show strong outward rotation by outwardly rotated feet^10,22^. Two trackways show evidence of increasing speed based on the increasing stride length and pace angulation (Supplementary Information Table S1). The trackway width is getting narrow in the trackways A and B because the hind limbs are more fully straightened as they attain a bipedal posture and a higher hip position^24,26^ (Fig. 4).

In trackway B, three successive manus prints (B1, B3, and B4) are preserved before the transition to bipedal locomotion. The lizard is the only vertebrate animal that starts on all limb pairs, then transitions to the bipedal gait by acceleration^23^. B1 has the best preservation amongst the three, which shows a complete manus imprint including metacarpal depression. On the other hand, B3 and B4 have only two or three incomplete distal digit imprints. The first bipedal stride by acceleration in lizards increases trunk angle, hence increases forelimb clearance^24^. The locomotor behaviour of the lizards that left trackways in the Hasandong Formation is similar to the experimental observations for the bipedal running of *Dipsosaurus dorsalis* and *Callisaurus draconoides*^27^. Therefore, consideration of all the evidence above strongly suggests that *S*. *hadongensis* was made by lizards transitioning to their hind limbs during locomotion and becoming bipedal.

**Figure 5:**
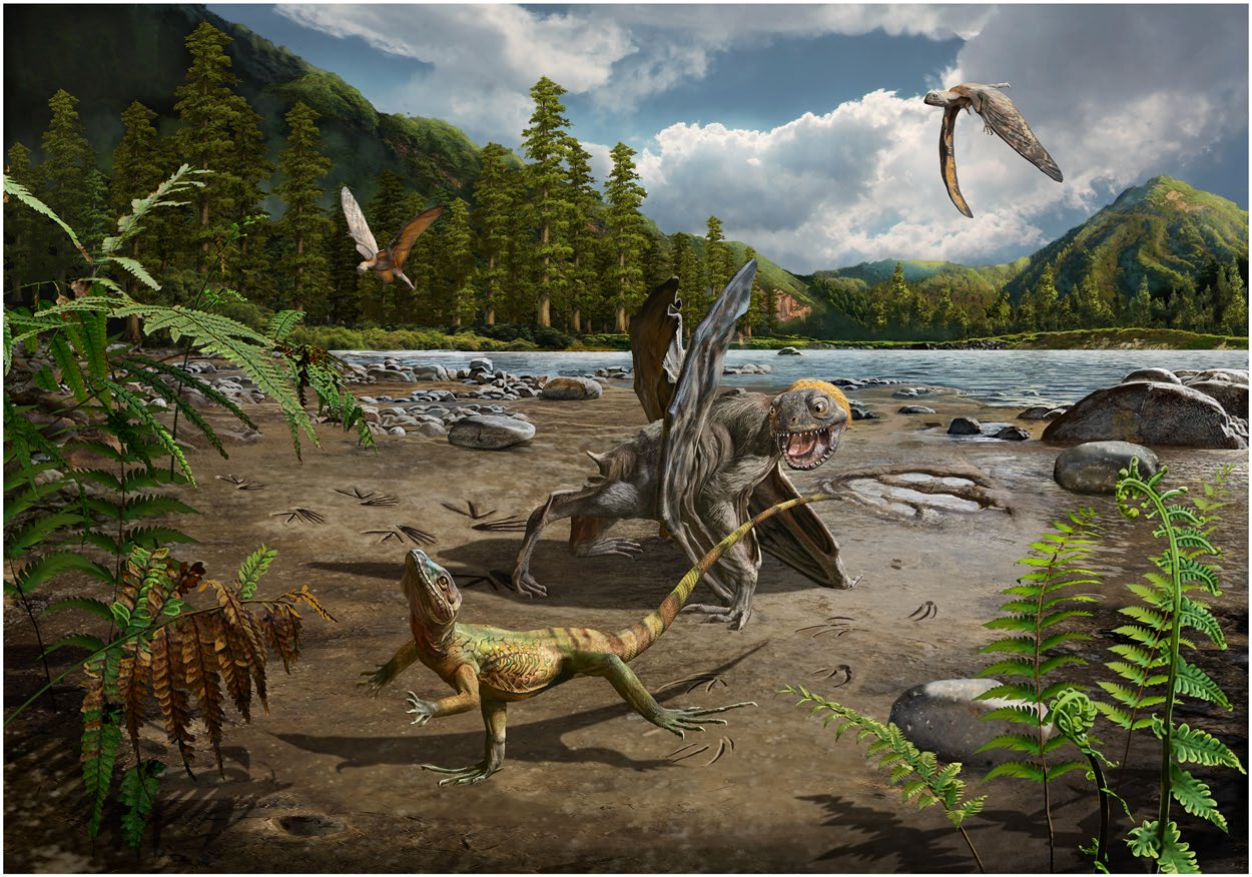
A reconstruction of a lizard running bipedally chased by the pterosaur *Pteraichnus koreanensis*, based on the trackway (Drawn by Chuang Zhao).

For the most part, bipedality has been related to fast locomotion and to predator avoidance^17^. Bipedality in lizards may be advantageous for enhanced environmental perception during locomotion by elevating the head and expanding the visual field during obstacle negotiation^28^. It is not certain whether *S*. *hadongensis* tracks were made when escaping from predators or not, but interestingly, the pterosaur track *Pteraichnus koreanensis* was reported from the same horizon at the same site^29^. Some pterosaurs likely foraged in diverse environments for small animals and carrion^30^. The occurrence of *P*. *koreanensis* and *S*. *hadongensis* tracks together may imply that these two trackmakers had a contemporary antagonistic relationship. If true, the threat of pterosaur predation might have caused these running lizards to leave the bipedal trackways found in the Hasandong Formation (Fig. 5).

### About the trackmaker

Bipedality can be observed in phylogenetically diverse extant lizard families such as Lacertoidea (teiids), Anguimorpha (varanids, bipedal posture), and Iguania (agamids, iguanids, crotaphytids, and phrynosomatids), particularly among the species that live in sandy, rocky or open environments^18,21^. The Gekkota was established in the Old World tropics by at least mid-Cretaceous^31^, and Late Jurassic basal gekkonomorphs (*Eichstaettisaurus schroederi* and *Ardeosaurus digitatellus*) already showed a capability for scansorial locomotion^32^. The Teiidae is native to the Americas^33,34^. In Asia, they first appeared in the Late Cretaceous in Mongolia and China^35^. Varanid lizards radiated from Mongolia during the Late Cretaceous to Early Cenozoic (80~50 Ma) and dispersed to almost all major fragments of Laurasia and Gondwana^36^.

Based on fossils and molecular data^2,37–39^, primitive iguanians (acrodontans and non-acrodontans) existed in Laurasia by the Aptian/Albian. Extant iguanians usually have well-developed, strong legs suitable for bipedality^21^. In addition, the extinct polyglyphanodonts are known from the Early Cretaceous in Asia^40^ and became abundant in the Upper Cretaceous of Mongolia and China^35,41^. They have strong hind limbs and a rather iguanian skeletal morphology. Therefore, based on the palaeobiogeographic distribution of facultative extant families, the lizard that produced *S*. *hadongensis* tracks could well have been a member of an extinct family or stem members of Iguania, which was present in the Early Cretaceous.

## Methods

### Geological Setting

The Hasandong Formation is inferred to be of Aptian to early Albian age based on a comprehensive paleomagnetic and radiometric data^9^. The overlying Jinju Formation and underlying Nakdong Formation have been dated to 109.9 ± 3.2 Ma and 127.67 ± 1.3 Ma, respectively^42,43^. The Hasandong Formation has yielded the most abundant vertebrate body fossils in the Gyeongsang Supergroup (Barremian~Campanian), part of the largest Mesozoic Gyeongsang Basin in the Korean Peninsula. Vertebrate fossils include turtles, pterosaurs, crocodilians, and dinosaurs^44^. Most bones occur as scattered, broken, and isolated pieces which had probably undergone long aerial exposure, transportation, and scattering on the floodplain before burial^45^. Previously described vertebrate ichnofossils from the Hasandong Formation include dinosaur tracks^46^ and pterosaur tracks of *Pteraichnus koreanensis*^29^.

The lizard track site is from an abandoned quarry next to the Hadong power plant, Hadong County where there is approximately 5,000 m^2^ of exposure, representing the middle part of the Hasandong Formation (Supplementary Information Fig. S1). The lizard trackways occur in the same horizon as the pterosaur ichnotaxon, *Pteraichnus koreanensis*, which comprises a dark grey mudstone layer in the middle part of the section. This layer also produces dinosaur tracks and plant fossils (*Ptilphylum* sp., *Cladophlebis* sp., *Ruffordia* sp.), presenting sediments thought to have been deposited in small swamps and/or marginal lakes associated with floodplains between channels.

## Electronic supplementary material

Supplementary Information

Supplementary Information Table 1
