# Supplementary material for: Lizards ran bipedally 110 million years ago
Source: Sci Rep. 2018 Feb 15;8:2617. doi: 10.1038/s41598-018-20809-z (PMC5814403; doi:10.1038/s41598-018-20809-z)
Supplement: Supplementary file 1 — Supplementary Information [file 41598_2018_20809_MOESM1_ESM.pdf]

Supplementary Information

Lizards ran bipedally 110 million years ago

Hang-Jae Lee[1], Yuong-Nam Lee*[,2], Anthony R. Fiorillo[3], and Junchang Lü[4]

[*]To whom correspondence should be addressed. E-mail: ynlee@snu.ac.kr

Supplementary Information

Supplementary Information References

Supplementary Information

Remarks on ichnotaxonomy of "lizards"

Rhynchosauroides from the Late Permian to the earliest Jurassic and *Ganasauripus* from the Late Permian are the only well-known ichnotaxa attributed to a "lacertoid" track [1-3]. However, most characteristics of the ichnogenus *Rhynchosauroides* are not seen in the walking tracks of any modern lizards [4]. Since the ichnogenus *Rhynchosauroides* was first named for the Early Triassic footprints from the Helsby Sandstone, United Kingdom [5], it has been reported in Europe, North America, and South America from Late Permian [1] to Late Jurassic [3]. The diagnosis of this ichnite has been emended by different authors [1,2,6-8]. The ichnogenus *Ganasauripus* was proposed for pentadactyl footprints of a quadrupedal reptile from Upper Permian Arenaria di Val Gardena Formation, Italy [1]. Valentini and others distinguished *Ganasauripus* from *Rhynchosauroides* because the latter shows a mesaxonic pes and an ectaxonic manus, while in *Ganasauripus* the pes is ectaxonic and the manus is mesaxonic [1]. However, a new morphological phylogenetic study on early saurian evolution indicates that there is no valid Permian record for Lepidosauromorpha [9]. The molecules and fossil data also support a Triassic origin for Lepidosauria [10]. If it is true, Permian *Rhynchosauroides* and *Ganasauripus* could have been made by primitive saurians rather than lepidosauromorphs. *Rhynchosauroides* is the most abundant and common in Triassic formations. The *Rhynchosauroides* trackmakers have been suggested to be lepidosauromorphs (Rhynchocephalia) or primitive archosauromorphs (Prolacertiformes), but no general agreement about its zoological affinity yet [7,11]. Rhynchocephalia, a sister clade of Squamata (lizards, amphisbaenians, snakes), became increasingly restricted to southern continents from the Middle Jurassic [12]. It is hypothesized that most major subclades within Squamata originated in the Cretaceous postdating major continental fragmentation [10].

Recently, a new ichnotaxon, *Neosauroides koreanensis*, was named for a lacertiform trackway from the Upper Cretaceous Haman Formation from Korea [13]. It was diagnosed by mesaxonic pentadactyl manus prints (N=4) smaller than the ectaxonic pes prints (N=3). However, all pes traces of *N. koreanensis* are incomplete consisting only of the distal traces of digits II, III, and IV, so that there is no whole pes print information. Though these tracks are incomplete, comparison of

these tracks with the tracks described in this study show significant differences. For example, the difference between the lengths of the five digits and digit divarication between digits of *N. koreanensis* are different from those of *Sauripes handongensis*.

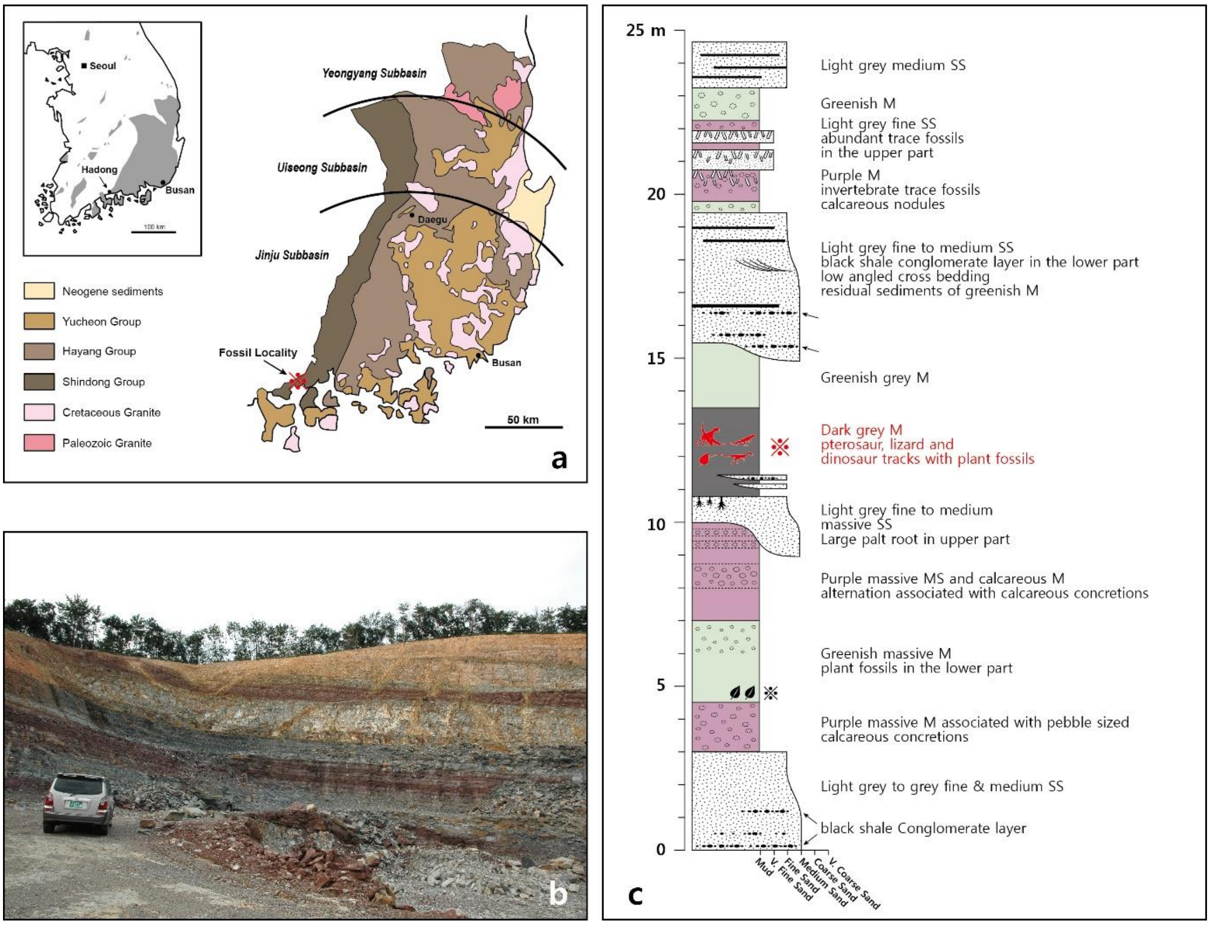

Supplementary Figure S1. Geological information of the fossil site**.** (**a)** Location map for the Lower Cretaceous lizard track locality in the Hasandong Formation, showing the position of the Cretaceous basins including the Gyeongsang Supergroup (modified from Lee et al., 2008) [14]. (**b)** Outcrop photograph of the fossil locality. (**c)** Geological section of the site indicating the lizard track-bearing level associated with pterosaur (*Pteraichnus koreanensis*) and theropod footprints, invertebrate traces, and plant fossils.

References

1. Valentini, M., Conti, M. A. & Mariotti, N. Lacertoid footprints of the Upper Permian Arenaria di Val Gardena Formation (Northern Italy). *Ichnos* **14,** 193–218 (2007).
2. da Silva, R. C., Ferigolo, J., de Souza Carvalho, I. & Fernandes, A. C. S. Lacertoid footprints from the Upper Triassic (Santa Maria Formation) of Southern Brazil. *Palaeogeogr. Palaeocl.* **262,** 140–156 (2008).
3. Avanzini, M., Pinuela, L. & García-Ramos, J. C. First report of a Late Jurassic lizard-like footprint (Asturias, Spain). *J. Iber. Geol.* **36,** 175–180 (2010).
4. Kubo, T. Extant lizard tracks: variation and implications for Paleoichnology. *Ichnos* **17,** 187–196 (2010).
5. Maidwell, F. Notes on footprints from the Keuper of Runcorn Hill. *Proc. Liverpool Geol. Soc.* **11,** 140–152 (1911).
6. Haubold, H. Therapsiden- und Rhychocephalen-Fährten aus dem Buntsandstein Südthüringens. *Hercinya* **3,** 147–183 (1966).
7. Haubold, H. Die tetrapodenfährten des Buntsandsteins in der deutschen Demokratische Republik und in Westdeutschland und ihre Aequivalente in der gesamten Trias. *Paläontol. Abh.* **4,** 421–439 (1971).
8. Klein, H. & Neidzwiedzki, G. Revision of the Lower Triassic tetrapod Ichnofauna from Wiory, Holy Cross Mountains, Poland. *NMMNH&S Bull.* **56,** 1–62 (2012).
9. Ezcurra, M. N. D., Scheyer, T. M. & Butler, R. J. The origin and early evolution of Sauria: reassessing the Permian saurian fossil record and the timing of the crocodile-lizard divergence. *PLoS ONE* **9,** e89165 (2014).
10. Jones, M. E. *et al.* Integration of molecules and new fossils supports a Triassic origin for Lepidosauria (lizards, snakes, and tuatara). *BMC Evol. Biol.* **13,** 1–1 (2013).
11. Avanzini, M. & Renesto, S. A review of *Rhynchosauroides tirolicus* Abel, 1926 ichnospecies (Middle Triassic: Anisian-Ladinian) and some inferences on *Rhynchosauroides* trackmaker. *Riv. Ital. Paleontol. S.* **108,** 51–66 (2002).

12. Evans, S. E., Prasad, G. V. R. & Manhas, B. K. Rhynchocephalians (Diapsida: Lepidosauria) from the Jurassic Kota Formation of India. *Zool. J. Linn. Soc.* **133,** 309–334 (2001).
13. Kim, K. S. *et al.* First report of lacertiform (lizard) tracks from the Cretaceous of Asia. *Cretaceous Res.* **69,** 62–70 (2017).
14. Lee, Y.-N., Lee, H.-J., Lü, J. & Kobayashi, Y. New pterosaur tracks from the Hasandong Formation (Lower Cretaceous) of Hadong County, South Korea. *Cretaceous Res.* **29,** 345–353 (2008).
